# Logistic Regression Model in a Machine Learning Application to Predict Elderly Kidney Transplant Recipients with Worse Renal Function One Year after Kidney Transplant: Elderly KTbot

**DOI:** 10.1155/2020/7413616

**Published:** 2020-08-19

**Authors:** Ubiracé Fernando Elihimas Júnior, Jamila Pinho Couto, Wallace Pereira, Michel Pompeu Barros de Oliveira Sá, Eduardo Eriko Tenório de França, Filipe Carrilho Aguiar, Diogo Buarque Cordeiro Cabral, Saulo Barbosa Vasconcelos Alencar, Saulo José da Costa Feitosa, Thais Oliveira Claizoni dos Santos, Helen Conceição dos Santos Elihimas, Emilly Pereira Alves, Marcio José de Carvalho Lima, Frederico Castelo Branco Cavalcanti, Paulo Adriano Schwingel

**Affiliations:** ^1^Programa de Pós-Graduação em Ciências da Saúde (PPGCS), Universidade de Pernambuco (UPE), Recife, PE 50100-130, Brazil; ^2^Unidade de Nefrologia e Divisão de Transplante, Universidade Federal de Pernambuco (UFPE), Recife, PE 50670-901, Brazil; ^3^Instituto de Ensino e Pesquisa Alberto Ferreira da Costa (IEPAFC), Real Hospital Português de Beneficência em Pernambuco (RHP/PE), Recife, PE 52010-075, Brazil; ^4^Unidade de Nefrologia, RHP/PE, Recife, PE 52010-075, Brazil; ^5^Serviço de Nefrologia, Hospital das Clínicas (HC), Universidade Federal de Pernambuco (UFPE), Recife, PE 50740-900, Brazil; ^6^Departamento de Fisioterapia, Universidade Federal da Paraíba (UFPB), João Pessoa, PB 58051-900, Brazil; ^7^Programa de Pós-Graduação em Engenharia de Sistemas (PPGES), UPE, Recife 50720-001, Brazil

## Abstract

**Background:**

Renal replacement therapy (RRT) is a public health problem worldwide. Kidney transplantation (KT) is the best treatment for elderly patients' longevity and quality of life.

**Objectives:**

The primary endpoint was to compare elderly versus younger KT recipients by analyzing the risk covariables involved in worsening renal function, proteinuria, graft loss, and death one year after KT. The secondary endpoint was to create a robot based on logistic regression capable of predicting the likelihood that elderly recipients will develop worse renal function one year after KT.

**Method:**

Unicentric retrospective analysis of a cohort was performed with individuals aged ≥60 and <60 years old. We analysed medical records of KT recipients from January to December 2017, with a follow-up time of one year after KT. We used multivariable logistic regression to estimate odds ratios for elderly vs younger recipients, controlled for demographic, clinical, laboratory, data pre- and post-KT, and death.

**Results:**

18 elderly and 100 younger KT recipients were included. Pretransplant immune variables were similar between two groups. No significant differences (*P* > 0.05) between groups were observed after KT on laboratory data means and for the prevalences of diabetes mellitus, hypertension, acute rejection, cytomegalovirus, polyomavirus, and urinary infections. One year after KT, the creatinine clearance was higher (*P* = 0.006) in youngers (70.9 ± 25.2 mL/min/1.73 m^2^) versus elderlies (53.3 ± 21.1 mL/min/1.73 m^2^). There was no difference in death outcome comparison. Multivariable analysis among covariables predisposing chronic kidney disease epidemiology collaboration (CKD-EPI) equation <60 mL/min/1.73 m^2^ presented a statistical significance for age ≥60 years (*P* = 0.01) and reduction in serum haemoglobin (*P* = 0.03). The model presented goodness-fit in the evaluation of artificial intelligence metrics (precision: 90%; sensitivity: 71%; and *F*_1_ score: 0.79).

**Conclusion:**

Renal function in elderly KT recipients was lower than in younger KT recipients. However, patients aged ≥60 years maintained enough renal function to remain off dialysis. Moreover, a learning machine application built a robot (Elderly KTbot) to predict in the elderly populations the likelihood of worse renal function one year after KT.

## 1. Introduction

Chronic kidney disease (CKD) is a worldwide public health problem [1]. There is an increase in treatment costs, as well as a worsening in the quality of life and psychosocial parameters [2]. There has also been a global increase in the number of patients with diabetes mellitus (DM), systemic arterial hypertension (SAH), and obesity, and these morbidities tend to progress with aging [3]. The last decade has witnessed a significant population growth, aging, and a fast pace of epidemiological study, with reduced mortality from communicable diseases and increased burden of noncommunicable diseases. Globally, DM and SAH were considered the two main comorbidities of CKD and increased significantly over in the last decades [4].

The World Health Organization for developing countries defines elderly person as being aged 60 years and over, and in developed countries, this chronological marking is defined from the age of 65 years [5]. Brazilian elderly statute considers elderly individuals aged 60 or over; however, from 2020 onwards, for social security, elderly Brazilian citizens aged 65 years or over for men and 63 years or over for women will be considered [6]. Brazil still finds it difficult to establish a balance between the aging of its population and financial cost of this new citizen in terms of health and social security [7].

In Brazil, the number in millions of people aged ≥60 years is set to increase from 19.6 in 2010 to 66.6 in 2050, thereby representing an increase of 239%. It is estimated that for every 21 elderly people in 2020, there will be 100 people in working age (18–59 years old). By 2050, this number will have risen to 51 elderly people for every 100 working-age persons [5]. Attention is also drawn to the growing rate of the world's elderly population. The evolution of medicine, prevention, and advances in health technology has led to increased life expectancy in several countries, such as Japan (83 years), Germany (80 years), United States (78 years), and Brazil (75 years) [8, 9].

Certain observations may be made regarding the change in the speed of economic development. Some countries, such as France, have had almost 150 years to adapt to a demographic transition from 10% to 20% in the proportion of the population over 60 years. Indeed, countries such as Brazil, China, and India will have just over 20 years in order to make the same adaptation [10].

Brazil also follows the worldwide trend of increase in the prevalence of end-stage renal disease (ESRD) and a rise in renal replacement therapy. According to the Brazilian Society of Nephrology (SBN), there were 42,695 patients on haemodialysis (HD) in 2000, equivalent to 503 per million population (pmp). These numbers have risen approximately 310% in 18 years with 133,000 patients on HD in 2018 (637 pmp), which is 44.3% of these aged 65 years and over [11].

Brazil has the largest publicly funded transplant program in the world and is the second country in absolute numbers in terms of kidney transplant (KT). However, the entire Brazilian KT system is exclusively financed by government taxes and managed by the Unified Health System (SUS, Portuguese) and free at the point-of-care, with all donations voluntary and altruistic [12]. KT improves the longevity and quality of life (QoL) of most patients with end-stage kidney disease (ESKD), including elderly patients in whom they are being increasingly performing renal replacement therapies (RRT) [2].

Donor agencies are a limited resource, and equitable KT allocation requires a balance between utility and justice. Similar to younger KT-recipients, donor quality affects the outcomes of elderly recipients and allografts. As a result, allocating more senile kidneys to older recipients can improve survival by reducing KT waiting time. In this sense, morbidity covariates such as malnutrition, depression, poor functional status, and social isolation can predict difficult post-KT follow-up. There is no consensus on a maximum age for transplanting CKD patients, but physiological age seems to be a better predictor of post-KT results and not chronological age [13]. Advanced chronological age alone should not prevent a patient from accessing KT, as current evidence suggests favourable elderly outcomes for both septuagenarians [14] and octogenarians [15, 16]. Therefore, after carefully considering the risks and benefits of KT, elderly people with CKD should continue to be considered for KT, with individualization on a case-by-case basis, rather than generalizations about therapeutic options based only on chronology [17].

KT for elderly recipients has comparable results of graft and patient survival when comparing elderly and young donors. Results of live donor transplantation in this age group are significantly better. Regardless, from practical considerations, our group supports the “old to old” program for kidney transplantation aiming maximizing the use of “expanded criteria” kidneys for aged recipients [18]. Studies have demonstrated that KT in elderly people is safe and successful, thereby improving the QoL and even doubling the survival time compared to elderly people on the transplantation waiting list [19]. Despite the demonstrated benefits of KT, when expertly referred, there are preestablished concepts that elderly people have a shorter life expectancy, present a higher surgical risk, and experience potential serious complications [20]. Results from the Scientific Registry of Transplant Recipients (SRTR) in the United States demonstrate that elderly patients on the waiting list experience significant survival benefit with KT [21]. The benefits are specially observed among patients whose life expectancy exceed 1.8 years and draw more attention in patients with ESRD caused by DM and/or SHT.

Analyzing the total of 659 KT carried out between January 2011 and July 2018 at Real Hospital Português de Beneficência em Pernambuco (RHP/PE) in Brazil, it was observed that 83 were patients aged ≥60 years, even though the service has no restrictions on the age of the recipient. Additionally, the number of transplants in elderlies at RHP/PE has not increased significantly and has remained stagnant near 12% over the years (approximately 10 KT by year). On the other hand, at this moment, 114 elderly patients are on the waiting list of the Brazilian National Transplant System (SNT) in the Pernambuco state and 5,786 in Brazil (Supplementary Materials SM1 and SM2 (available here)). Also, Brazilian population is aging and nearly 45,000 patients aged ≥65 years are undergoing RRT only on SUS in 2018 [11].

Due to these epidemiological data presented, our group was motivated to carry out a study to demonstrate and draw attention to the elderly transplanting possibility even in patients with low economic and social status. The Gini Brazil index was 0.53 in 2018 [22], and in the state of Pernambuco, the Gini index was 0.63 in 2010 [23], with a per capita income of US$ 204.00 (March 19, 2020) [24, 25]. Even with this socioeconomic disparity, there are still benefits to transplantation in the elderly in terms of establishing renal function capable of removing them from HD therapy. In addition, demonstrating that chronological age alone should not be part of the criteria that exclude elderly kidney patients from the waiting list for KT patients.

In this sense, the present study has two complementary objectives. The primary endpoint was to compare elderly (≥60 years) versus younger (18 to 59 years) KT recipients by analyzing the risk covariables involved in worsening renal function, proteinuria, graft loss, and death one year after KT. Secondary endpoint was to create a robot based on the logistic regression model capable of predicting the likelihood that elderly KT recipients will develop worse renal function one year after KT (chronic kidney disease epidemiology collaboration (CKD-EPI) < 60 mL/min/1.73 m^2^).

## 2. Materials and Methods

### 2.1. Study Design and Assessed Data

A unicentric retrospective cohort study conducted with the medical records (MR) of patients aged ≥18 years undergoing KT from January to December 2017 at RHP/PE, Brazil. All patients selected from MR were followed for one year after KT. MR with unavailable information or of patients who abandoned treatment were excluded.

**Figure 1:**
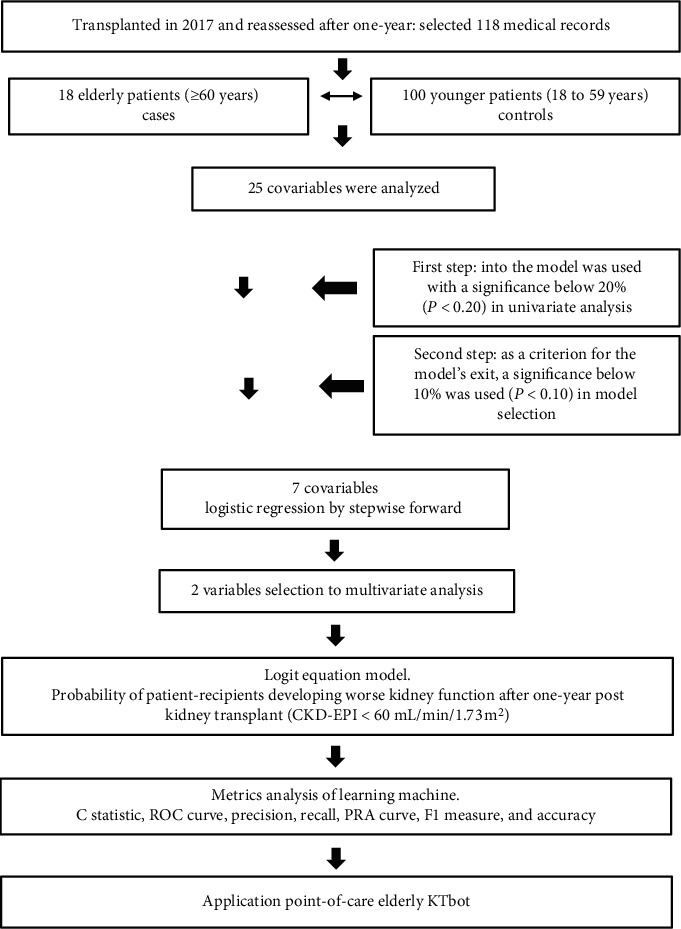
Flowchart with the selection of individuals in the retrospective cohort, research design, and strategy to build the Elderly KTbot.

Patients were divided into two groups: elderly (aged ≥60 years or the case group) and younger (aged 18–59 years or the control group). The patient selections and study design are presented in Figure 1.

Data were catalogued and included in an online platform and encrypted to maintain the confidentiality and security of the database. After structuring, data were exported to a Structured Query Language (SQL)™ relational database. All data accessed by the group were cryptographed and kept confidential until the statistical approach was carried out. Finally, the dataset has been decrypted and reconfigured for interpretation in Microsoft Excel 2016™ (Microsoft Corporation, Redmond, USA, Release 12026.20320, 2019).

The study complied with the Helsinki Declaration (1964) and is in agreement with the Resolutions 466/2012 and 510/2016 of the Brazilian National Health Council. The Ethics Committee on Human Research from HUOC/PROCAPE at the Universidade de Pernambuco, Brazil, approved the study (CAAE: 82587418.6.0000.5192). All data were kept confidential.

### 2.2. Immunosuppression Protocol Used by RHP/PE

Patients at high immunological risk received 2 mg/kg of thymoglobulin in three consecutive doses on days zero, three, and six plus 1 g of methylprednisolone at induction, followed by an initial maintenance with 0.2 mg/kg/day of tacrolimus, 1440 mg/day of sodium mycophenolate, and 0.5 mg/kg of prednisone with a maximum dose of 30 mg in the first week, followed by a reduction of 5 mg per week to a dose of 5 mg/day. High immunological risk was defined as retransplantation, panel-reactive antibody (PRA) of 50% or higher, or the presence of donor-specific antibody (DSA) with a mean fluorescence intensity of between 1500 and 5000.

Patients at low immunological risk were induced with a single dose of thymoglobulin at 3 mg/kg and 1 g of methylprednisolone, followed by initial maintenance with 0.1 mg/kg/day of tacrolimus, 3 mg/day of sirolimus, or 3 mg/day of everolimus and 0.5 mg/kg of prednisone with a maximum dose of 30 mg in the first week, followed by a reduction of 5 mg per week to a dose of 5 mg/day. Low immunological risk was PRA below 50% in first KT with no DSA.

Tacrolimus was adjusted to a serum level between 4 and 8 ng/mL (0.004–0.008 *μ*g/mL) in high-immune risk recipients and between 3 and 5 ng/mL (0.003–0.005 *μ*g/mL) in low-immune risk recipients. Everolimus and sirolimus were adjusted to levels between 3 and 6 ng/mL (0.003 *μ*/mL–0.006 *μ*/mL).

Kidneys from deceased donors aged ≥60 years or between 50 and 59 years, with two of the following criteria: (a) serum creatinine ≥1.5 mg/dL or 114.38 *μ*mol/L; (b) history of SAH; (c) death from stroke, referred to as expanded criteria donors (ECD) were allocated only to patients over 50 years of age with 0% PRA (nonsensitized recipients). This strategy is used to increase the allocation of kidneys to the elderly patients.

### 2.3. Covariables Analysed

Twenty-five covariables linked to pre- and post-KT were analysed subdivided in (a) general covariables (sex, race, age, obesity, RRT modality, and time on RRT pre-KT); (b) comorbidities covariables pre-KT (SAH and DM); (c) transplant immunological covariables (donor type, panel-reactive antibody (PRA), rejection, immunosuppression, mismatch in locus human leukocyte antigen DR (HLA-DR), HLA-A, HLA-B, presence of DSA, and new-onset diabetes after transplant (NODAT)); (d) infection covariables (polyomavirus (PV), cytomegalovirus (CMV) infection, and urinary tract infection (UTI)); (e) laboratory serum covariables (haemoglobin, phosphorus, calcium, albumin, and creatinine); (f) laboratory urinary covariables (proteinuria >0.3 g/24 h and microalbumin); (g) renal function variable (glomerular filtration rate (GFRe) estimated by the CKD-EPI one year after KT); and (h) clinical outcome of the recipient covariables (maintained transplant, death, and returned to RRT).

### 2.4. Statistical Analysis

Dataset was decrypted before interpretation by the statistical package and then categorized and prepared for analysis. The confidentiality of the database was permanently maintained, and statistical analysis was performed using R Project™ (R Foundation for Statistical Computing™, Vienna, Austria, Release 3.4.2, 2019). Initially, descriptive statistics were applied to the Kolmogorov–Smirnov test and to Bartlett's criteria. Continuous covariables were expressed as mean ± standard deviation (minimal-maximal values), while categorical covariables were presented in frequencies and percentages. Associations among variables were assessed using Pearson's chi-square test or Fisher's exact test. Student's *t*-test and the Mann–Whitney *U* test were used to compare case and control groups. All statistical methods were two-tailed, confidence intervals (CI) were exact, and the significance level was set to *P* < 0.05.

### 2.5. Logistic Regression Model and Algorithms of Machine Learning

For estimates of adjusted associations, univariate analysis was used. The logistic regression model was applied, in which the adjusted association measures were estimated, and association measures were presented using odds ratio (OR) [26].

In the study, a statistical tool of logistic regression was used as a parametric to model, interpret, and handle the binary or multinomial responses of the data evaluated as a preprocessing step for machine learning. The model can be expressed by

(1) $$\begin{array}{c} \ln\left(\frac{\pi }{1-\pi }\right)=\sum_{i=0}^{k}\beta_{i}x_{i}⟺\pi =\frac{e^{\beta_{0}+\sum_{i=1}^{k}\beta_{i}x_{i}\;}}{1+\;e^{\beta_{0}+\sum_{i=1}^{k}\beta_{i}x_{i}\;}}, \end{array}$$

where *π* is the probability of the event of the elderly patient recipients developing worse kidney function after one year post-KT, *x*_*i*_ is the *i*^th^ covariate of classification of the response, and *β*_*i*_  is the *i*^th^ coefficient of logarithmic regression [27].

Stepwise forward applies the logistic modelling method, and the criterion of entry into the model was used with *P* < 0.20 in the univariate analysis, and *P* < 0.10 was used with a criterion for the model's exit. In all stages of the modelling, biological plausibility was considered, being the basis for all interpretations of the concomitances.

### 2.6. Practical Application for the Model in Point-of-Care

In order to predict the probability of the allograft receptor developing renal function estimated by CKD-EPI < 60 mL/min/1.73 m^2^ in the one year post-KT, the regression coefficients were presented in the logit equation form. In this manuscript, we developed a learning machine application using software in Microsoft Windows Form application for Borland C++, NET Framework™ (Microsoft Corporation, Redmond, USA), named Elderly Kidney Transplant Bot (Elderly KTbot). This supplementary material and the main source codes are available for free at https://doi.org/10.6084/m9.figshare.12043404 for other centres to be able to reproduce the mathematical prediction tool.

## 3. Results

### 3.1. General Demographic Data of the Study Population

A total of 118 patients underwent KT with a mean age of 45.6 ± 12.8 (18–75) years, and 51.7% was male. Of these, 18 (15.3%) were aged ≥60 years and composed the elderly group (case group), and 100 (84.7%) were younger patients (control group). The mean age of the elderly group (65.0 ± 4.4 years) was higher (*P* < 0.001) than the younger group (42.0 ± 10.4 years).

The number of elderly patients presented with a previous diagnosis of DM before KT (relative risk (RR): 8.1; CI_95%_: 2.5–25.7) was greater than in the youngers (*P* < 0.001). Furthermore, the elderly group had a higher frequency of ECD kidneys, and no recipients of this group received a living donor KT (Table 1). There was no statistical difference between the groups regarding the frequencies of all infectious covariables (PV, CMV, and UTI), acute rejection, and SAH. Moreover, the frequency of NODAT was also similar between groups (17% in the control group and 6% in the case group).

### 3.2. Immunologic Covariables

The occurrence of two mismatches in the HLA-A locus (*P* < 0.05) was higher in the elderly group (66.7%) than in the younger group (35.4%). There was no difference between groups regarding PRA and the presence of DSA (Table 2).

In the assessment of laboratory tests in the first year after KT (Table 3), only increased levels of proteinuria were more prevalent in the elderly than in younger ones (RR: 3.9, CI_95%_: 1.3–11.7).

The combined assessment of proteinuria predisposing factors (>0.3 g/24 h) such as DM before KT and NODAT in KT recipients and the use of immunosuppressants (prednisone and inhibitors of mammalian target of rapamycin (mTOR)) demonstrated that only a history of DM before KT was the risk factor for proteinuria (OR: 20.7; IC_95%_: 3.6–119.6). None of proteinuria-related covariates were significant for probability of the renal dysfunction model (Table 4).

Renal function assessment analysed by the estimation of creatinine clearance (CrCl) using CKD-EPI equation presented statistical difference between the two groups (Table 5). The best renal function was observed in the younger group with CrCl mean (70.9 ± 25.2) higher (*P* < 0.01) than in the elderly group (53.3 ± 21.1).

### 3.3. Multivariate Analysis by Stepwise Forward

Table 6 describes univariate and multivariate analyses of risk factors for CKD-EPI < 60 mL/min/1.73 m^2^. Two covariables demonstrated significance among the risk factors predisposing to a lower CKD-EPI: age ≥60 years (*P* = 0.01) and serum haemoglobin (*P* = 0.03).

**Figure 2:**
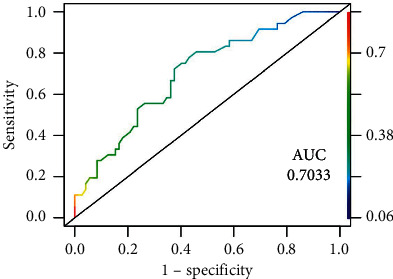
Logistic regression modelling (ROC curve) among the risk factors for kidney function (CKD-EPI) < 60 mL/min/1.73 m^2^ one year after transplanting.

Figure 2 presents the statistic C and area under the ROC curve (AUC) equal to 0.70 estimated by the logistic regression modelling for receiver operating characteristics among the risk factors for CKD-EPI < 60 mL/min/1.73 m^2^ one year after KT.

**Figure 3:**
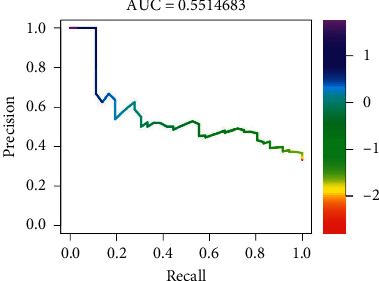
Precision and recall curve of logistic regression modelling for probabilistic estimator for CKD-EPI < 60 mL/min/1.73 m^2^ one year.

Precision-recall of logistic regression modelling for probabilistic estimators for CKD-EPI < 60 mL/min/1.73 m^2^ one year after KT is presented in Figure 3.

Table 7 presents the evaluation metrics of logistic regression modelling with a 90% of precision. Sensibility is identified as 71% and *F*_1_ score as 79%.

### 3.4. Final Model Resulted in the following Probability Equation

(2) $$\begin{array}{c} \pi \left(\text{CKDEPI}<\frac{60\;\text{mL}/\min}{1.73\;{\text{m}}^{2}}\right)=\;\frac{1}{1+e^{-\left(3.06+1.54\times {\text{Age}}_{\geq 60\text{ years}}-0.31\times \text{Hb}\right)}}, \end{array}$$

where *e*=2.71 (Napier's constant or Euler's number), age_≥60 years_ = 1 if age ≥60 years or = 0 if age < 60 years, Hb: serum haemoglobin,*π* = probability, and CKDEPI = filtration rate value using CKD-EPI equation from serum creatinine [28].

## 4. Discussion

### 4.1. Biological Plausibility of the Model

The increased prevalence of CKD is associated with the aging population in developed countries, with an increasing number of patients on a waiting list and a higher frequency of KT in elderly people [29]. It has been demonstrated that elderly transplant patients have a better survival rate in comparison to elderly people on a waiting list [30, 31]. Increasing the acceptance of deceased donors, using ECD organs, has become a strategy to increase the number of KT and to reduce the number of patients on HD [31]. According to Knoll et al. [13], the decrease in mortality compared to patients on HD may range from 41% to 60%.

The number of ECD in KT has increased over recent years, even though the survival rate is lower than that of kidneys from standard criteria deceased donors (SCDD) (16). In the present study, patients from both groups were transplanted predominantly with kidneys from SCDD (83.3% and 85.0% in elderly and younger groups, respectively). There was a higher frequency (*P* = 0.021) of patients in the elderly group (16.7%) transplanted with a kidney from an ECD in relation to the younger group (3.0%).

Immunosuppression protocol used by RHP/PE allocates ECD kidneys primarily to patients aged 50 years and elderly, based on studies that demonstrate a lower survival of ECD grafts [32, 33]. Moreover, while 12% of the KT performed on the younger group was living donor transplants, none of the transplants in the elderly group was performed with a graft from this type of donor. Most elderly patients received the kidney from standard deceased donors in a similar way in despite the immunosuppression protocol adopted.

DM is a multifactorial disease, and its prevalence increases with advancing age, exceeding 10% in patients aged over 60 years [34]. All patients in the present study were immunosuppressed with a regimen that included tacrolimus and prednisone. Both of these drugs are known to induce DM [29], especially during the first six months when they are used in higher doses. Calcineurin inhibitors [31] may cause NODAT with an incidence rate of up to 20.5% [32]. This diabetogenic effect seems to be influenced by aging. NODAT may occur at a relative risk of 90% in patients aged between 45 and 59 years and can reach 160% in recipients aged 60 years and over [29]. Glucocorticoids are involved through increased insulin resistance and hepatic gluconeogenesis. Calcineurin inhibitors, especially tacrolimus, induce a defect in insulin secretion, interfering with the activation of pancreatic beta cells [31]. In spite of this, there was no higher incidence of NODAT among elderly patients, which occurred in one patient (6%), when compared to younger patients. It is possible that the lower number of cases in the elderly group may have influenced this result.

Mismatch analysis demonstrates a statistically significant increase in HLA-A locus incompatibilities in elderly patients. Perhaps, this result may express only a higher population prevalence of the antigen. No biological plausibility was found for such statistical result, and it was also not evidenced in multivariate analysis.

The influence of proteinuria on the progression of kidney disease is well-known in nephrology [35, 36]. A higher occurrence (*P* = 0.017) of proteinuria (>0.3 g/24 h) was observed in the elderly recipients (56.3%) in comparison to the younger group (24.7%). The aetiology of proteinuria may be related to mTOR inhibitors, chronic graft dysfunction, calcineurin inhibitor nephrotoxicity [37], and diabetic nephropathy [29]. Posttransplant proteinuria is an independent risk factor for cardiovascular risk and a recognized cause of loss of renal function [38]. In the combined analysis of proteinuria and risk factors for proteinuria after KT, we observed that the prevalence of DM in the pre-KT phase was the most statistically significant factor for proteinuria in the elderly group. This result corroborates the need to further intensify the approach to combat DM during the pre-KT phase. Although in the logistic regression, proteinuria has not shown a significant role for the decline kidney function on CKD-transplant patients one year after KT. For this reason, proteinuria was not used in construction of the Elderly KTbot. In addition, the Elderly KTbot modelling demonstrated that proteinuria is not important in the former for renal function of the elderly. Despite this, it does not mean that proteinuria should not be monitored and treated.

Finally, the results of logistic regression show that the probability of having a worse kidney function one year after KT is higher for the elderly and lower for patients with higher haemoglobin. Elderly patient has a 4.67 greater chance of having worse kidney function one year after KT when compared to young patients. On the other hand, the chance of a person having worse kidney function is 1.35 greater for a decrease of one unit of haemoglobin in serum haemoglobin levels.

With regard to renal function one year after KT, the elderly group presented with a mean lower level of CrCl than the younger group but with adequate reserve to remain off RRT. This result is consistent with previous data [39] where levels of CrCl in young KT patients are higher in relation to the outcomes (survival and renal functions) when compared to the elderly patients in the long-term. Nevertheless, as previously discussed, transplantation in elderly patients outweighs the risks related to remaining on HD [40].

In Brazil, Gouveia et al. [41] demonstrated that when compared to the cost of HD modalities, treatment becomes cost-effective after two years of successful KT. The number of KT performed in Brazil is still below the needs of the growing waiting list, including for elderly patients. In 2018, 5,923 KT were performed, equivalent to just 28.8 pmp. Additionally, 81.5% of the 5,486 KT on adults was performed on patients aged between 18 and 59 years, while only 18.4% was on patients aged 60 years and over [12]. Data from SBN reported that 34.3% of patients on chronic HD aged 65 years and over in 2017 [12].

### 4.2. Kidney Transplantability in the Elderly

There is no formal transplantation barrier for elderly patients. It is not the chronological age that determines the condition of patient but, rather, patient's performance status. Baby boomers are growing elderly, and therefore, becomes elderly boomers. Concerns regarding the “excess” of children are giving way to the “excess” of elderly people. It is necessary to learn to deal with the aging population [42].

In this context, monitoring and following appropriate risk stratification, preoperative protocols, and pre-KT consultations should be directed towards patient transplantability. Most elderly patients are not placed on the list of recipients simply because they are not even referred for pre-KT consultations. It may even be suggested that prejudice and involuntary discrimination are thus being exercised, based on the age of patients, presenting a genuine social barrier against elderly people, and thereby a reflection of ageism [43, 44].

For elderly people, “aging associated with RRT is a daily challenge in the search for quality and time of life” [45]. Within this context, they may be victims of ageism, a term coined in 1969 by Robert Neil Butler, in an article published in The Gerontologist, to identify discriminatory practices against elderly people, including institutional and political intolerances. These positions may perpetuate stereotypes about elderly people, with little regard for their individual competences [43, 46].

According to data from the Brazilian Ministry of Health provided and extracted from the Brazilian SNT until December 2019, of the 25,163 patients on the waiting list for KT, only 2911 (9.5%) were aged 60 years or over (Supplementary Material SM2). This proportion varies according to the region of the country, from 11.5% in the Midwest to 19.7% in the Southeast, directly proportional to the per capita income and life expectancy of the region. Although this difference may be related to the historical coexistence of socially active elderly individuals in more developed regions [5]. Brazilian population, as in other countries, is in the process of aging, and this phenomenon is irreversible [8, 47]. Efforts should be made to increase the frequency of KT in this population, and in this sense, our results corroborates others [48] and demonstrated that it is possible to transplant elderly people without damage.

### 4.3. Ageism, New Concepts, and New Best Practices on Elderly Patients

Ageism (current term) leads to the exclusion of elderly people within their communities. It is everywhere and perhaps the most “normalized” form of prejudice, since, unlike racism or sexism, it is not widely opposed [44]. Due to this perspective, elderly people are denied work and are restricted to social services and stereotyped in the media [41]. Brazil needs to learn from other countries to see elderly as an integral and active part of society [8].

However, learning to deal with the geriatric patient should be part of the current situation in all countries that are experiencing the same aging characteristics of Brazil. In recent years, several guidelines have been developed to assess the patient globally in surgical procedures. Multiprofessional teams in satellite clinics should be trained to use geriatric and frailty screening and start to understand considerations of applications of preoperative frailty scores in clinical practice. In case of doubt or lack of training, the transplant centre should be able to assess and apply the geriatric performance assessment scores [49]. What should be avoided is the impossibility of elderly patient to have access to the benefit of KT. By virtue of insufficiency or absence of technical knowledge about age and aging [50], advanced age should not be an absolute contraindication for KT in carefully selected patients with ESRD. KT offers a higher survival coefficient than HD treatment even in the elderly. Another point that should be taken into account is elderly recipients on the waiting list have a higher mortality rate when compared to KT recipients of similar age [51].

According to this context, new concepts on geriatric patient assessment have been proposed by the societies of surgeons and anaesthesiologists. On the base of evidence from the preoperative evaluation of the elderly are the well-established forecast surgical outcomes including complications, length of stay, functional dependence, and death [52]. A comprehensive geriatric assessment offers a much more exhaustive picture of the individual physiological fitness and functional reserves in comparison with traditional evaluation. According to the guidelines [52–56] and principles of geriatric domain assessment, a minimum preoperative assessment for the elderly should include functional assessment for activities of daily living [57], instrumental activities of daily living [58], neurocognitive [59], mini-mental state exam [60], and psychoaffective screening [61], nutritional status [62], comorbidity cumulative illness rating scale for geriatrics [63], identification of potentially inappropriate medications, risk of postoperative delirium, risk of fall, availability of social support, and frailty.

In summary, clinicians, nephrologists, and multiprofessional teams should be able to include elderly patients on the waitlist of KT recipients, assessing their clinical, psychic, and social conditions. The path to transplantation in elderly people, therefore, depends on their being viewed as socially active. It is necessary to replace ageism with new guidelines and preoperative geriatrics best practices in the evaluation of geriatric population. Age is not a risk factor for rejection in the first year. Elderly should be given the right to receive a KT or at least be registered on the waiting list. Even with socioeconomic inequalities, it is still possible to offer quality medicine to the population. The principles present in many National Health Systems are universality (everyone should have the right) and equity (equal treatment for equals and unequal treatment for unequal's), and those who need more help should have more support. That is, the main point is the elderly needs to be treated with more equity both in the indication of the KT and in the inclusion of the elderly in the waiting list of the KT.

### 4.4. Logistic Regression Multivariate Modelling, Machine Learning, and Small Data

Logistic regression modelling by stepwise forward is able to identify with a degree of precision and expressive criticality and the probability of elderly recipients to develop renal function CKD-EPI < 60 mL/min/1.73 m^2^ one year after KT. The model that gives rise to the Elderly KTbot has a precision (positive predictive value) of 90% and a recall (sensitivity) of 71%, as it combines the harmonic average of precision and recall.

The *F*_1_ measure score is considered one of the best discriminants of the model's power artificial learning and was 0.76 in this research. The value closer to 1 demonstrates greater predictive power of the machine learning model. It is worth mentioning that despite the limited number of patients coming from the waiting lists, the result of the centre was quite expressive without deaths in the elderly up to 1-year-olds and with the ability to remain in outpatient conservative therapy.

The main point of the study is to demonstrate a way to study its population of transplant recipients and using methodologies that combine clinical experience, biological plausibility, and the learning machine. It is possible to create tools to aid in prediction and bedside treatment. The construction of robots can help to understand the dynamic behaviour of the data, even in small unbalanced groups and with restricted numbers of samples. Artificial intelligence is capable of handling both big data [64] and small data [65], provided that supervised and unsupervised learning processes have human participation. Clinical thinking is able to enhance the fundamental and explanatory random covariates that relate to the primary outcomes [65].

### 4.5. Limitations

It is a single-centre cohort using secondary data and restricted number of elderly patients. This fact is probably related to nonreferral of elderly to pretransplant consultations, which consequently leaves them out of the waiting list for KT. This study tries to demonstrate not only the possibility but also the social need to register elderlies on the waiting list. In this sense, our group guaranteed confidentiality through encrypted platforms and presented a model that can be reproduced as a predictive tool for the elderly.

In addition, the research used a predominantly mixed population of Brazilian Amerindians, multiracial (afro descendants), descendants of Germans, Dutch, British, Jews, Arabs, and Portuguese according to the characteristics of colonization [66], and phylogeography of Pernambuco state of Brazil [67]. On the other hand, the introduction of probabilistic metrics for machine learning analysis increased the safety of the developed multivariate model, as well as enhances the safety of using the Elderly KTbot for elderly KT populations.

## 5. Conclusions

Kidney allograft function estimated by CKD-EPI equation was higher in patients aged <60 years. Elderly patients did not present a higher risk of death one year after KT, and multivariate analysis demonstrated that the probability of having a worse kidney function one year after KT is higher for the elderly and lower for patients with higher haemoglobin. The logistic regression model proved to be precise and sensitive and can be used in the day-to-day medical decision.

Because KT behaves as an extremely complex nonlinear and nonparametric system with a large number of variables known and unknown, any tool capable of assisting the medical decision is welcome. In this sense, the nephrology service from RHP/PE and postgraduation programs in systems engineering (PPGES) and in health sciences (PPGCS) at the University of Pernambuco in combination provided free of charge an Elderly KTbot capable to predict in the elderly populations the likelihood of worse renal function one year after KT.

## Figures and Tables

**Table 1:** Pretransplant comorbidity history among younger and elderly patients.

Pre-KT covariables	<60 years (*n* = 100)	≥60 years (*n* = 18)	RR	95% CI	*P*
	*n* (%)	*n* (%)			
Time on RRT pre-KT, months	50.5 ± 46.1 (0–258)	49.6 ± 33 (8–108)	—	—	0.675^*∗∗∗*^
Obesity pre-KT			1.046	0.316–8.722	
No	93 (93%)	16 (88.9%)			0.625^*∗∗*^
Yes	07 (7%)	02 (11.1%)			
Systemic arterial hypertension pre-KT			3.594	0.778–16.596	
No	31 (31%)	02 (11.1%)			0.084^*∗*^
Yes	69 (69%)	16 (88.9%)			
Diabetes mellitus pre-KT			8.089	2.549–25.667	
No	91 (91%)	10 (55.6%)			0.001^*∗∗*^
Yes	09 (09%)	08 (44.4%)			
Type of donor			—	—	
Expanded spectrum of deceased donor	03 (03%)	03 (16.7%)			0.021^*∗*^
Standard criteria deceased donor	85 (85%)	15 (83.3%)			
Living donor	12 (12%)	00 (-)			

RR: relative risk; CI: confidence interval; RRT: renal replacement therapy; pre-KT: before kidney transplant. ^*∗*^Chi-square test; ^*∗∗*^Fisher's exact test; and ^*∗∗∗*^ Mann–Whitney *U* test.

**Table 2:** Comparison of prekidney transplant immunological covariables between younger and elderly patients one year after kidney transplantation.

Immunologic covariables	<60 years (*n* = 100)	≥60 years (*n* = 18)	*P*
	*n* (%)	*n* (%)	
PRA			
0%	65 (68.4%)	11 (61.1%)	0.800^*∗*^
<50%	11 (11.6%)	03 (16.7%)	
50–79%	06 (6.3%)	02 (11.1%)	
>80%	13 (13.7%)	02 (11.1%)	
Mismatch in HLA DR			
0	28 (31.1%)	10 (55.6%)	0.120^*∗*^
1	48 (53.3%)	07 (38.9%)	
2	14 (15.6%)	01 (5.6%)	
Mismatch in HLA A			
0	12 (12.5%)	01 (5.6%)	0.046^*∗*^
1	50 (52.1%)	05 (27.8%)	
2	34 (35.4%)	12 (66.7%)	
Mismatch in HLA B			
0	15 (15.6%)	01 (5.6%)	0.522^*∗*^
1	35 (36.5%)	07 (38.9%)	
2	46 (47.9%)	10 (55.6%)	
DSA			
No	85 (85%)	14 (77.8%)	0.487^*∗∗*^
Yes	15 (15%)	04 (22.2%)	

PRA: panel-reactive antibody; HLA: human leukocyte antigen; DSA: donor-specific antibody. ^*∗*^Chi-square test; ^*∗∗*^Fisher's exact test; and ^*∗∗∗*^ Mann–Whitney *U* test.

**Table 3:** Comparison of laboratory covariables between younger and elderly patients one year after kidney transplantation.

Laboratory covariables	<60 years (*n* = 100)	≥60 years (*n* = 18)	*P*
	*n* (%)	*n* (%)	
Serum			
Haemoglobin (g/dL)	13.2 ± 1.8 (9.1–19); *n* = 92	13.2 ± 1.6 (9.3–15.7); *n* = 16	0.988^*∗∗∗∗*^
Phosphorus (mg/dL)	3.5 ± 3.8 (1.3–33); *n* = 66	3.1 ± 0.6 (2.1–4.1); *n* = 12	0.983^*∗∗∗*^
Calcium (mg/dL)	9.3 ± 2.1 (1.1–13.9); *n* = 65	9.9 ± 0.9 (8.8–11.6); *n* = 12	0.683^*∗∗∗*^
Albumin (g/dL)	4.3 ± 0.6 (2.9–7.2); *n* = 43	4.5 ± 0.1 (4.4–4.6); *n* = 4	0.242^*∗∗∗*^
Urinary			
Proteinuria >0.3 g/24 h			
No	70 (75%)	07 (44%)	0.017^*∗∗*^
Yes	23 (25%)	09 (50%)	
Microalbumin			
<30 mg/dL	07 (25%); *n* = 28	00 (-); *n* = 4	0.209^*∗*^
30 to 300 mg/dL	06 (21.4%); *n* = 28	00 (-); *n* = 4	
>300 mg/dL	15 (53.6%); *n* = 28	04 (100%); *n* = 4	

PRA: panel-reactive antibody; HLA: human leukocyte antigen; DSA: donor-specific antibody. ^*∗*^Chi-square test; ^*∗∗*^Fisher's exact test; ^*∗∗∗*^ Mann–Whitney *U* test; and ^*∗∗∗∗*^ Student's *t*-test.

**Table 4:** Analysis of composite outcomes for postkidney transplant proteinuria >0.3 g/24 h among younger and elderly risk patients.

Covariables	<60 years (*n* = 100)	≥60 years (*n* = 18)	OR	95% CI	*P* ^*∗*^
	*n* (%)	*n* (%)			
Proteinuria			3.913	1.310–11.689	
No	70 (75.3%)	07 (43.8%)			0.017
Yes	23 (24.7%)	09 (56.3%)			
Total	93 (100%)	16 (100%)			
Prekidney transplant urine protein + DM pre-KT			20.68	3.576–119.627	
No	91 (97.8%)	11 (68.8%)			0.001
Yes	02 (2.2%)	05 (31.3%)			
Total	93 (100%)	16 (100%)			
Postkidney transplant urine protein + NODAT			0.819	0.094–7.144	
No	86 (92.5%)	15 (93.8%)			1.000
Yes	07 (7.5%)	01 (06.3%)			
Total	93 (100%)	16 (100%)			
Urine protein + mTOR inhibitor			1.111	0.283–4.353	
No	77 (82.8%)	13 (81.3%)			1.000
Yes	16 (17.2%)	03 (18.8%)			
Total	93 (100%)	16 (100%)			
Urine protein + mTOR inhibitor + corticosteroid			1.111	0.283–4.353	
No	77 (82.8%)	13 (81.3%)			1.000
Yes	16 (17.2%)	03 (18.8%)			
Total	93 (100%)	16 (100%)			
Urine protein + mTOR inhibitor + corticosteroid + calcineurin inhibitor			1.111	0.283–4.353	
No	77 (82.8%)	13 (81.3%)			1.000
Yes	16 (17.2%)	03 (18.8%)			
Total	93 (100%)	16 (100%)			

OR: odds ratio; CI: confidence interval; KT: kidney transplant; DM: diabetes mellitus; NODAT: new-onset diabetes after transplant; and mTOR: mammalian target of rapamycin. mTOR inhibitor was everolimus or sirolimus. Calcineurin inhibitor was cyclosporine or tacrolimus. Corticosteroid was prednisone. ^*∗*^Chi-square test.

**Table 5:** Comparison between the recipient renal function with the CKD-EPI and clinical outcomes after the first year of kidney transplantation amongst the younger and older patients.

Covariables	<60 years (*n* = 100)	≥60 years (*n* = 18)	*P*
	*n* (%)	*n* (%)	
Serum creatinine (mg/dL)	1.2 ± 0.5 (0.5–3.8)	1.6 ± 0.6 (0.9–2.8)	0.020^*∗∗∗*^
Renal function by CKD-EPI equation	70.9 ± 25.2 (0–121.6)	53.3 ± 21.1 (21.1–91.9)	0.006^*∗∗*^
Clinical outcome of the recipient			
Functioning allograft	98 (98.0%)	17 (94.4%)	0.051^*∗*^
Death	00 (-)	01 (5.6%)	
Returned to RRT	02 (2.0%)	00 (-)	

CKD-EPI: chronic kidney disease epidemiology collaboration; RRT: renal replacement therapy; ^*∗*^Chi-square test; ^*∗∗*^Student's *t*-test; and ^*∗∗∗*^ Mann–Whitney *U* test.

**Table 6:** Univariate and multivariate analysis of risk factors for CKD-EPI < 60 mL/min/1.73 m^2^ one year after kidney transplant.

Univariate analysis					Multivariate analysis			
Covariables	OR crude (95% CI)	OR adjusted (95% CI)	*β* (SE)	*P*	Covariables	OR adjusted (95% CI)	*β* (SE)	*P*
DM pre-KT	0.69 (0.20–2.35)	0.69 (0.18–2.21)	−0.37 (0.62)	0.56				
Standard deceased donor								
Yes	—	—	—	—				
No	1.58 (0.57–4.37)	1.58 (0.56–4.35)	0.46 (0.52)	0.37				
Mismatch HLA-A								
0	—	—	—	—				
1	1.84 (0.44–7.65)	1.84 (0.48–9.06)	0.61 (0.73)	0.40				
2	1.34 (0.31–5.80)	1.34 (0.34–06.80)	0.30 (0.75)	0.69				
Proteinuria >0.3 g/24 h	1.07 (0.45–2.56)	1.07 (0.44–2.53)	0.07 (0.45)	0.88	Constant	—	3.06 (1.72)	
Age ≥ 60 years	4.23 (1.40–12.83)	4.23 (1.43–13.57)	1.44 (0.57)	0.01	Age ≥ 60 years	4.67 (1.52–15.55)	1.54 (0.58)	0.01
Haemoglobin^*∗*^	—	1.69 (1.04–1.32)	−0.28 (0.12)	0.03	Haemoglobin	1.35 (1.06–1.78)	−0.31 (0.13)	0.03

CKD-EPI: chronic kidney disease epidemiology collaboration; DM: diabetes mellitus; KT: kidney transplant; HLA: human leukocyte antigen; OR: odds ratio; CI: confidence interval; *β*: beta model coefficient; SE: standard error; multivariate analysis model: *P* < 0.002; Chi-square test (2 degrees of freedom): 12.64; determination coefficient (*R*^2^) of Nagelkerke: 0.15; C statistics: 0.70; and area under the curve (AUC): 70%. ^*∗*^Crude odds ratio for haemoglobin was not calculated.

**Table 7:** Metrics to evaluate the performance (power) of the multivariate regression stepwise model and the robot named Elderly Kidney Transplant Bot (Elderly KTbot).

Metric machine learning or probability estimator	Value
Precision	0.9028
Recall or sensibility	0.7143
F1 measure or *F*_1_ score	0.7975
Accuracy	0.6944

## Data Availability

The authors confirm that the data supporting the findings of this study are available within in the supplementary materials. The Elderly KTbot (Supplementary Material) is available for free in figshare at https://doi.org/10.6084/m9.figshare.12043404, reference number 12043404.

## Supplementary Materials

https://doi.org/10.6084/m9.figshare.12043404 Supplementary Materials The authors confirm the addition of three supplementary materials: SM1: table with the total number of patients aged ≥60 years waiting for kidney transplantation in the waitlist of the Brazilian National Transplant System presented according to the, respectively, federative unit of Brazil. SM2: table 2 with consolidated data of the number of patients aged ≥60 years waiting for kidney transplantation in the waitlist of the Brazilian National Transplant System presented between 2011 and 2019. Elderly KTbot: application created using logistic regression modelling to identify the probability that kidney recipients of kidney transplant will develop an estimated renal function <60 mL/min/1.73 m^2^ one year after transplantation. We also provide the source code of programming performed in Borland C++ in Microsoft Visual Studio called Windows Forms App^TM^ (.NET framework). The application and the source codes are available from https://doi.org/10.6084/m9.figshare.12043404.
