# Supplementary material for: Logistic Regression Model in a Machine Learning Application to Predict Elderly Kidney Transplant Recipients with Worse Renal Function One Year after Kidney Transplant: Elderly KTbot
Source: J Aging Res. 2020 Aug 19;2020:7413616. doi: 10.1155/2020/7413616 (PMC7453245; doi:10.1155/2020/7413616)
Supplement: https://doi.org/10.6084/m9.figshare.12043404 Supplementary Materials — The authors confirm the addition of three supplementary materials: SM1: table with the total number of patients aged ≥60 years waiting for kidney transplantation in the waitlist of the Brazilian National Transplant System presented according to the, respectively, federative unit of Brazil. SM2: table 2 with consolidated data of the number of patients aged ≥60 years waiting for kidney transplantation in the waitlist of the Brazilian National Transplant System presented between 2011 and 2019. Elderly KTbot: application created using logistic regression modelling to identify the probability that kidney recipients of kidney transplant will develop an estimated renal function <60 mL/min/1.73 m2 one year after transplantation. We also provide the source code of programming performed in Borland C++ in Microsoft Visual Studio called Windows Forms AppTM (.NET framework). The application and the source codes are available from https://doi.org/10.6084/m9.figshare.12043404. [file 7413616.f1.docx]

Supplementary Material SM1: Total number of patients aged ≥ 60 years waiting for kidney transplantation in the waitlist of Brazilian National Transplant System presented according to the respectively federative unit of Brazil

| Federative unit of Brazil | Total number of patients | Number of elderlies (aged ≥ 60 years) | % of elderlies in the waitlist |
| --- | --- | --- | --- |
| MT | 0 | 0 | 0.00% |
| PI | 232 | 5 | 2.16% |
| AC | 25 | 2 | 8.00% |
| PE | 1.012 | 114 | 11.26% |
| MS | 122 | 14 | 11.48% |
| DF | 551 | 68 | 12.34% |
| BA | 1.290 | 160 | 12.40% |
| AL | 284 | 36 | 12.68% |
| MA | 249 | 35 | 14.06% |
| RN | 265 | 39 | 14.72% |
| PB | 286 | 43 | 15.03% |
| MG | 3.243 | 523 | 16.13% |
| SC | 477 | 77 | 16.14% |
| ES | 1.156 | 188 | 16.26% |
| PR | 1.540 | 255 | 16.56% |
| CE | 943 | 158 | 16.76% |
| PA | 385 | 65 | 16.88% |
| GO | 247 | 44 | 17.81% |
| RS | 1.277 | 229 | 17.93% |
| RJ | 1.620 | 312 | 19.26% |
| RO | 106 | 22 | 20.75% |
| SP | 14.403 | 3.397 | 23.59% |
| Total general | 29.713 | 5.786 | 19.47% |

Data Source: Computerized Management System. General Coordination of National Transplant System. Ministry of Health. Brazil. Date of extraction: August 31, 2019.

AC: Acre; AL: Alagoas; AM: Amazonas; BA: Bahia; CE: Ceará; DF: Distrito Federal; ES: Espírito Santo; GO: Goiás; MA: Maranhão; MG: Minas Gerais; MT: Mato Grosso; MS: Mato Grosso do Sul; PA: Pará; PB: Paraíba; PE: Pernambuco; PI: Piauí; PR: Paraná; RJ: Rio de Janeiro; RN: Rio Grande do Norte; RO: Rondônia; RS: Rio Grande do Sul; SC: Santa Catarina; SE: Sergipe; SP: São Paulo.

Supplementary Material SM2: Consolidated data number of patients aged ≥ 60 years waiting for kidney transplantation in the waitlist of Brazilian National Transplant System presented between 2011 and 2019

| Federative unit of Brazil | 2011 | 2012 | 2013 | 2014 | 2015 | 2016 | 2017 | 2018 | 2019 |
| --- | --- | --- | --- | --- | --- | --- | --- | --- | --- |
| AC | 0 | 1 | 0 | 0 | 3 | 5 | 2 | 1 | 1 |
| AL | 22 | 12 | 16 | 20 | 33 | 29 | 32 | 5 | 7 |
| AM | 120 | 46 | 23 | 18 | 14 | 10 | 1 | 0 | 0 |
| BA | 85 | 80 | 57 | 25 | 33 | 36 | 52 | 91 | 90 |
| CE | 35 | 63 | 60 | 56 | 53 | 52 | 76 | 89 | 105 |
| DF | 12 | 10 | 26 | 26 | 33 | 24 | 32 | 27 | 36 |
| ES | 39 | 74 | 69 | 53 | 67 | 67 | 58 | 78 | 93 |
| GO | 18 | 18 | 18 | 13 | 10 | 15 | 22 | 32 | 47 |
| MA | 12 | 10 | 14 | 23 | 7 | 12 | 11 | 9 | 19 |
| MG | 107 | 142 | 150 | 157 | 155 | 143 | 155 | 220 | 257 |
| MS | 6 | 28 | 29 | 0 | 1 | 1 | 4 | 9 | 11 |
| MT | 38 | 14 | 75 | 7 | 0 | 0 | 0 | 0 | 4 |
| PA | 84 | 68 | 154 | 103 | 83 | 77 | 59 | 38 | 35 |
| PB | 17 | 16 | 26 | 22 | 23 | 23 | 39 | 19 | 25 |
| PE | 69 | 47 | 40 | 81 | 70 | 46 | 72 | 89 | 95 |
| PI | 24 | 20 | 27 | 42 | 45 | 9 | 49 | 2 | 1 |
| PR | 79 | 136 | 130 | 148 | 134 | 138 | 180 | 177 | 222 |
| RJ | 61 | 40 | 85 | 106 | 138 | 121 | 155 | 143 | 187 |
| RN | 13 | 7 | 8 | 16 | 21 | 22 | 19 | 21 | 32 |
| RO | 0 | 0 | 0 | 3 | 9 | 5 | 3 | 5 | 10 |
| RS | 116 | 157 | 134 | 161 | 159 | 158 | 150 | 171 | 174 |
| SC | 53 | 61 | 55 | 56 | 49 | 62 | 61 | 64 | 99 |
| Brazil | 1.871 | 1.852 | 1.896 | 1.984 | 2.023 | 2.230 | 2.520 | 2.627 | 2.911 |

Data Source: Computerized Management System. General Coordination of National Transplant System. Ministry of Health. Brazil. Date of extraction: February 17, 2020.

AC: Acre; AL: Alagoas; AM: Amazonas; BA: Bahia; CE: Ceará; DF: Distrito Federal; ES: Espírito Santo; GO: Goiás; MA: Maranhão; MG: Minas Gerais; MT: Mato Grosso; MS: Mato Grosso do Sul; PA: Pará; PB: Paraíba; PE: Pernambuco; PI: Piauí; PR: Paraná; RJ: Rio de Janeiro; RN: Rio Grande do Norte; RO: Rondônia; RS: Rio Grande do Sul; SC: Santa Catarina; SE: Sergipe; SP: São Paulo.
